# Camel plasma as an innovative non-antibiotic therapy for *Escherichia coli*-induced colibacillosis in neonatal lambs: a comparative pilot study with antibiotics, camel milk, and probiotics under a One Health framework

**DOI:** 10.14202/vetworld.2026.1132-1148

**Published:** 2026-03-17

**Authors:** Hussein Radhi Tuama, Nameer A. Khudhair, Mohanad Faris Abdulhameed

**Affiliations:** Department of Public Health, College of Veterinary Medicine, University of Basrah, Basrah province, Iraq

**Keywords:** antimicrobial resistance, camel milk, camel plasma, colibacillosis, *Escherichia coli*, neonatal lambs, non-antibiotic therapy, One Health

## Abstract

**Background and Aim:**

Neonatal colibacillosis caused by *Escherichia coli* is associated with high mortality in lambs, resulting in significant economic losses and contributing to concerns about antimicrobial resistance (AMR). Conventional treatment relies heavily on antibiotics, but non-antibiotic alternatives are urgently needed to reduce AMR and support passive immunity transfer. This pilot study evaluated camel plasma as a novel non-antibiotic therapeutic option for *E. coli* infection in neonatal lambs, comparing its efficacy with antibiotics, camel milk, and commercial probiotics, while aligning with One Health principles to promote sustainable livestock production and reduce zoonotic risks.

**Materials and Methods:**

Rectal swab samples from 10 naturally diarrheic lambs were initially collected and subjected to bacteriological culture on MacConkey and Eosin methylene blue agars, followed by polymerase chain reaction (PCR) targeting the *uidA* gene (162 base pair amplicon) for species confirmation. Antimicrobial susceptibility was determined using the Kirby–Bauer disk diffusion method against five antibiotics. Twenty diarrheic lambs (≤ 2 weeks of age, Awassi breed) were randomly assigned to four treatment groups (n = 5 each) for a 5-day intervention: antibiotics (gentamicin and ciprofloxacin administered intramuscularly), camel milk (5 mL/kg orally twice daily), camel plasma (5 mL/kg subcutaneously daily), and probiotics (5 × 10^9^ colony-forming units orally twice daily). Clinical parameters (appetite, hydration, fecal consistency, activity), hematological values (white blood cell [WBC] count, granulocytes, red blood cells, etc.), biochemical parameters (total protein, albumin, globulin, creatinine, liver enzymes), and serum immunoglobulin G (IgG) and immunoglobulin A (IgA) concentrations (measured by enzyme-linked immunosorbent assay [ELISA]) were assessed before and after treatment. The physicochemical properties of camel milk were also determined. Data were analyzed using one-way analysis of variance followed by Tukey’s honestly significant difference test (p < 0.05).

**Results:**

All isolates were confirmed as *E. coli*, showing 40%–60% susceptibility to the tested antibiotics. Camel milk composition averaged 3.29% fat, 3.83% protein, and 5.80% lactose. The camel plasma group exhibited the most pronounced clinical improvements, including markedly better appetite, activity, hydration status, and fecal consistency (returning to soft/pasty), with no adverse reactions observed. All treatments significantly reduced WBC counts (from 14.78 ± 3.60 to approximately 7 × 10^9^/L) and granulocyte counts (from 10.98 ± 3.26 to approximately 4 × 10^9^/L; p < 0.05). Biochemical parameters such as albumin, total protein, globulin, and creatinine showed moderate increases in the camel plasma group. ELISA results demonstrated significant stabilization of IgG levels (from approximately 5 to 2 μg/mL; p = 0.001) in the camel milk, camel plasma, and probiotic groups, with the most notable normalization of IgA occurring in the camel plasma group (from 2.03 ± 0.43 to 0.42 ± 0.15 μg/mL; p < 0.05).

**Conclusion:**

Camel plasma demonstrated superior therapeutic efficacy compared with antibiotics, camel milk, and probiotics in improving clinical signs, modulating inflammatory responses, and supporting passive immunity in neonatal lambs with colibacillosis. As a promising non-antibiotic intervention, camel plasma has the potential to reduce antimicrobial use, limit the spread of AMR, enhance farm biosecurity, and decrease the risk of zoonotic transmission in resource-limited settings. These findings strongly support further large-scale, long-term studies to validate safety, optimize dosing, and explore broader applications within a One Health framework.

## INTRODUCTION

Neonatal diarrhea is a critical health issue that leads to significant economic losses. A high mortality rate in young lambs is associated with inadequate passive transfer of maternal immunity and exposure to infectious pathogens, including rotavirus, *Escherichia coli*, *Salmonella* spp., and *Cryptosporidium* spp. [1]. Among these, *E. coli* infection is the most prevalent in lambs aged less than 2 weeks. This bacterium produces several deadly toxins, including Shiga toxins (produced by Shiga toxin-producing *E. coli* [STEC]), enterotoxins (produced by enterotoxigenic *E. coli* [ETEC]), EhxA, and necrotizing *E. coli* toxins (NTEC) [2]. Transmission occurs mainly via the fecal–oral route due to farm mismanagement, poor sanitation, and herd congestion. Lambs with colibacillosis exhibit various clinical signs, including watery diarrhea, dehydration, and lethargy [3]. Mortality rates vary with seasonal factors and management practices [4]. Inadequate colostrum feeding in newborn lambs reduces performance and increases susceptibility to infection. Antibiotic treatment may not be feasible due to individual immunodeficiency and antimicrobial resistance (AMR) [5].

Suckling lambs primarily rely on immunoglobulin absorption from colostrum secreted by the mammary glands of ewes, with absorption possible for 3–6 days after birth. Antibodies must be absorbed within the first 6–12 h of life, a critical window for achieving passive immunity transfer and establishing defense mechanisms against opportunistic pathogens [6]. Colostrum is a nutrient-rich fluid providing both immunological and nutritional support, mainly composed of immunoglobulin G (IgG), lactose, proteins, minerals, and fatty acids. Mortality may exceed 50% in newborn lambs with failure of passive transfer (FPT), with incidence ranging from 3.4% to 20% [7]. Lambs with passive immunity failure are more susceptible to bacterial or viral infections, experience higher mortality, and show impaired growth. Failure of passive immunity (FPI) is associated with risk factors including insufficient colostrum intake, low-quality colostrum, and poor management [8]. Low colostrum quality and quantity may result from environmental stress, poor hygiene, and inadequate dietary energy intake by dams during gestation. Survival rates decline between 24 and 72 h of life markedly if serum gamma-globulin concentration is below 0.5 g/100 mL [9, 10].

Several studies on *E. coli* isolates from sheep in Iraq have demonstrated notable variation in AMR, multidrug resistance (MDR), and the prevalence of extended-spectrum β-lactamases (ESBLs). *E. coli* has shown resistance to multiple drugs, including ampicillin, amoxicillin, chloramphenicol, gentamicin, and ciprofloxacin, with susceptibility percentages varying across regions [11, 12]. Various AMR genes, including CTX-M, SHV, TEM, rfbo157, and fliCH7, have been detected in ESBL-producing isolates from sheep in northern and central Iraq [13, 14]. *E. coli* also harbors mobile genetic elements known as integrons, which act as gene cassettes that capture and carry multiple antibiotic resistance genes [15]. These resistance patterns not only limit therapeutic options for managing neonatal diarrhea in lambs but also pose a public health concern through zoonotic transfer of resistance genes from animals to humans [16]. The presence of MDR- and ESBL-producing *E. coli* highlights the urgent need for selective antibiotic use, improved farm hygiene, and control strategies such as annual immunization and regular probiotic supplementation.

Efforts to reduce mortality among newborn lambs have focused on alternative therapeutic approaches, including plant-derived compounds, bacteriophages, probiotics/prebiotics, and artificial rearing with milk replacers. Commercial probiotics are commonly added to neonatal lamb feed, providing benefits such as enhanced immune function, improved growth rates, and reduced bacterial infections. However, frequent probiotic use carries a risk of horizontal gene transfer (HGT) between pathogenic and non-pathogenic bacteria [17, 18]. A few studies have explored camel milk as a means to address various health issues in humans and animals. Camel milk contains abundant bioactive enzymes, immunoglobulins, and minerals with anti-inflammatory and antioxidant properties [19, 20]. It has been reported as an effective supportive therapy in conditions such as diabetes, asthma, cancer, and hypertension [21]. Although camels adapt well to desert environments, their remote migration in search of food and water can limit milk availability during lactation. Treatment of diarrheic lambs and calves has traditionally relied on supportive care and antibiotics. Recent research has emphasized antimicrobial peptides (AMPs) due to their rapid bactericidal activity and immunomodulatory effects [22, 23]. However, their clinical use is limited by susceptibility to enzymatic degradation, poor pharmacokinetics, and low bioavailability [24].

Despite growing evidence of AMR in *E. coli* isolates from sheep, particularly MDR and ESBLs, effective non-antibiotic strategies for treating neonatal colibacillosis remain limited [11–16]. While probiotics, camel milk, and AMPs have been investigated as alternatives, they face constraints, including potential HGT, variable bioavailability, sourcing difficulties, and inconsistent field performance [17–24]. Passive immunotherapy using plasma or serum has been documented in neonatal ruminants for FPT, but its therapeutic application specifically against *E. coli*-induced diarrhea in lambs has received little attention. Camel plasma, characterized by heavy-chain antibodies with superior antigen-binding affinity and exosome-mediated immunomodulation, represents a promising yet untested intervention in this context [25]. To date, no controlled field studies have directly compared camel plasma with conventional antibiotics, camel milk, and probiotics in naturally infected neonatal lambs, nor have they evaluated its impact under a One Health lens to simultaneously address animal health, AMR reduction, farm biosecurity, and zoonotic risk mitigation. This critical research gap restricts the adoption of sustainable, immunity-supporting therapies in resource-constrained farming systems.

Most previous studies have compared the effects of antibiotics or probiotics in ruminants, or have used camel milk experimentally to improve biochemical parameters and animal performance as supportive nutrition [26–28]. In contrast, the present study offers a unique and innovative side-by-side comparison of four therapeutic strategies in the same clinical setting: antibiotics, camel milk, camel plasma, and probiotics. This direct evaluation of four therapeutic modalities in naturally infected diarrheic lambs provides a novel comparative framework that has not been previously reported.

The present study aimed to bridge this gap by assessing camel plasma as a novel non-antibiotic therapeutic agent for *E. coli* infection in neonatal lambs. Through a comparative pilot field trial, the efficacy of camel plasma was evaluated against antibiotics, camel milk, and probiotics in terms of clinical recovery (appetite, activity, hydration, fecal consistency), hematological parameters (WBC count, granulocytes), biochemical profiles (total protein, albumin, globulin, creatinine, liver enzymes), and serum immunoglobulin levels (IgG and IgA). The overarching objective was to determine whether camel plasma could effectively neutralize pathogens, bolster passive immunity, and improve lamb survival while reducing reliance on antimicrobials, thereby supporting AMR mitigation and aligning with One Health principles to enhance livestock welfare, economic sustainability, and public health safety in arid and semi-arid regions.

## MATERIALS AND METHODS

### Ethical approval

Ethical approval for this study was obtained from the Scientific and Ethical Committee of the College of Veterinary Medicine, University of Basrah, Iraq (Approval No. 85/37/2025, dated January 4, 2024). All procedures involving animals were conducted in strict accordance with the institutional guidelines for the care and use of animals in research and complied with international standards set by the World Organization for Animal Health, Animal Research: Reporting of *In Vivo* Experiments 2.0 guideline, and the recommendations of the International Society for Applied Ethology. Written and verbal informed consent was obtained from all sheep and camel owners prior to the commencement of the study after explaining the research objectives, sampling procedures, possible risks, and therapeutic interventions. Participation was voluntary, and farmers retained the right to withdraw their animals at any time.

A licensed veterinarian handled all lambs to minimize discomfort, stress, and pain. The following welfare protocols were adhered to: animals were restrained using gentle, non-invasive methods appropriate for neonates. All blood and fecal samples were collected using sterile techniques by certified veterinary personnel. To prevent anemia and stress, needle sizes and sampling volumes were selected in accordance with OIE guidelines for neonates. Dehydrated lambs were monitored twice daily, and when necessary, supportive care (oral electrolytes, warming, and hydration) was provided. Severe dehydration or non-responsiveness was treated with immediate supportive therapy according to humane standards.

### Study period and location

The study was conducted from January to April 2025 in the Safwan subdistrict, located in the southwestern region of the Basrah Governorate, approximately 51 km from Basrah city. The Safwan region lies close to Kuwait and has arid climatic conditions, especially in summer. The temperature during the winter months is usually between 10°C and 18°C, while in the summer it is extremely hot, averaging 50°C. The geographical coordinates of the study area are approximately 30°08′N latitude and 47°03′E longitude. This location represents an extensive rearing zone for small ruminants during winter and spring, particularly during the lambing season. Camels are also a dominant species in this region, where the Bedouin tribe owns camels for socio-economic purposes and has a cultural legacy.

### Animal sampling

Convenient sampling was adopted to select the animals for this study. A total of 30 suckling lambs (≤ 2 weeks of age, weight 3–4 kg) descended from the Awassi (Naimi) breed were included. These animals exhibited clear symptoms of watery diarrhea and were selected from two farms. The selected animals exhibited dehydration, with a score ≤ 5%. Lambs with severe dehydration (score ≥ 10%) and extreme fatigue were described as having bad prognoses and an inability to withstand the period of study, and were excluded. Ten lambs were used for initial bacterial identification, and the remaining 20 lambs were incorporated in the field-based study under real farm conditions (Basrah, Iraq). The identities of farms and owners were kept confidential. The first author is an MSc student and a licensed veterinarian who diligently carried out this work to achieve the research program in collaboration with local animal farmers.

### Microbiological testing methods

Rectal swab samples were assembled directly from diarrheic lambs using a sterile cotton swab. The samples were collected in the morning, preserved in sterile containers, and then transferred by ice box to the Bio-Vet Laboratory (a Private Microbiological and Veterinary Lab located in the center of Basrah city). The team of lab workers consisted of four people (two technicians and two supervisors—physiologists and microbiologists) who assisted with sample analysis. The samples were subjected to bacteriological and antibiotic susceptibility tests. The samples were cultured on nutrient agar and transferred to MacConkey and Eosin methylene blue agar. The media were incubated for 24 h at 37°C. The isolates were identified as suspected *E. coli* based on their morphological features. The molecular method was used to identify the bacterial species. Antibiotic susceptibilities were determined for colonies using the disk diffusion (Kirby–Bauer) test. A panel of five antibiotics, including amoxicillin, norfloxacin, ciprofloxacin, gentamicin, and chloramphenicol, was tested. The zones of inhibition were measured using diameter-based breakpoints, which categorized antibiotic sensitivity as susceptible, intermediate, and resistant, according to the laboratory guideline developed by the Clinical and Laboratory Standards Institute (CLSI) M100 [29]. Upon completion of the study, all biological wastes were autoclaved before disposal.

### Camel milk collection

Before collecting the milk samples, the udder and teats were cleaned thoroughly with clean water and then dried. Teats were disinfected with 70% alcohol. The first foremilk was discarded and decanted in a sterilized container. Fresh camel milk (50–100 mL) was collected from eight clinically healthy, well-managed adult lactating camels. The milk samples were stored in sterile containers at 4 °C until use. 5 mL of milk from each animal was analyzed using the Lactoflash Milk Analyzer (Funk Gerber, Berlin, Germany) to determine the physicochemical properties (fat and solid-non-fat [SNF], relative density [RD], freezing point [FP], protein, and lactose).

### Camel plasma collection

No sedation was required, and the camels were manually restrained to avoid stress. Camels’ blood (N = 8) was collected from the same camels that were milked. Blood samples were directly obtained from the jugular vein using a 50 mL disposable syringe (18G × 1-1/2) and placed in sterilized containers containing ethylene-diamineetetraacetic acid. Camels were monitored for bleeding, swelling, or discomfort after collection. The samples were transferred to the laboratory and distributed into plain tubes for centrifugation at 4000 rpm for approximately 10 min to harvest the camel plasma. Subsequently, they were stored in the freezer refrigerator (−18°C). Plasma was used exclusively for research animals and was not returned for human or commercial use.

### Probiotic characterization

A commercial probiotic product (Biolact/BioActiveT, United Kingdom) was used in this study. The formulation was specifically asformulated for young animals and contains various strains of lactic acid bacteria, including *Lactobacillus helveticus*, *L. acidophilus*, *L. bulgaricus*, and *Streptococcus thermophilus*, in well as three other important strains: *Bifidobacterium bifidum*, *B. infantis*, and *B. longum*. Beneficially, these non-pathogenic bacteria can sustain the digestive function of the alimentary tract, inhibit other pathogenic microorganisms, and boost immunity. Each sachet contained a probiotic concentration of 5 × 10^9^ CFU and was administered orally after being mixed with milk/water.

### Study design for a field-based therapeutic study

A small-scale pilot study was conducted to evaluate the feasibility and efficacy of administering a new treatment/immunity enhancer, including camel milk and camel plasma, to neonate lambs infected with colibacillosis. The study involved random allocation of 20 sick lambs into four groups of five animals each (5 replicates per group). The lambs were sourced from two farms (each with 10 selected animals), where they were housed on the same farms and remained with their dams in a semi-enclosed field. These animals were supplied with adequate food and water ad libitum, with a circular water-drinking trough (1 m diameter × 0.7 m depth) and a feed basin (1 m length × 0.6 m width × 0.4 m height). The housing environment was cleaned daily to maintain hygienic conditions.

### Clinical treatment in a field study

**Figure 1:**
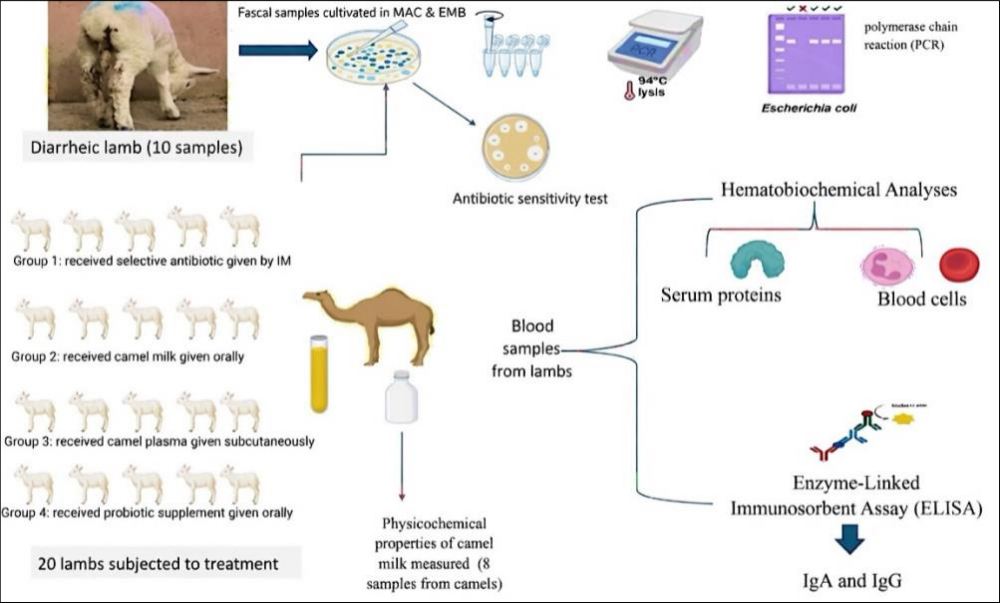
Design and treatment trial implications (BioRender.com).

The 20 diarrheic lambs received four treatment types according to the designed groups (Figure 1). Lambs were monitored daily for clinical signs, body temperature, fecal consistency, and dehydration. Fecal samples were collected from each animal before treatment and then subjected to bacteriological and molecular diagnoses. Blood samples were collected aseptically before and after treatment. Approximately 4 mL of blood was drawn directly from the jugular vein of each lamb. One milliliter was placed in a sterilized test tube containing ethylenediaminetetraacetic acid for hematological analyses, while the remaining 3 mL was decanted into a gel-containing tube. The samples were transferred to the laboratory for hematological, biochemical, and immunological analyses, and subjected to the enzyme-linked immunosorbent assay (ELISA) to evaluate IgG and IgA levels.

At the beginning of the study, three lambs in the antibiotic group that died due to severe pre-existing weakness were immediately reported to the ethical committee and replaced to maintain group balance. All deaths were recorded, and carcasses were disposed of in accordance with biosafety protocols. They were replaced immediately with three other animals to maintain an equal group size and complete the final database for inclusion criteria. Each treatment group is described as follows:

- Group 1 (Antibiotics Group): Lambs received selective antibiotic therapy based on the results of the disk diffusion sensitivity test. Gentamicin 10% (1 mL/10 kg body weight [BW]) and ciprofloxacin 10% (1 mL/20 kg BW) were both administered intramuscularly (IM) twice a day. The administered dose was based on the manufacturer’s instructions.
- Group 2 (Camel Milk Group): Treated orally by feeding camel milk mixed with dam milk at a dose of 5 mL/kg twice a day. The volume was increased to 20 mL to compensate for spillage during consumption. Dose = 5 mL/kg × BW (average BW of lamb was approximately 3 kg; dose = 15 mL and increased to 20 mL).
- Group 3 (Camel Plasma Therapy Group): Replicates were administered 5 mL/kg of camel plasma subcutaneously. The total injected dose was 15 mL, considered a safe dose for enhancing FPT, every day for 5 days. The dose was validated based on previous studies of blood and plasma transfusions in ruminants [30, 31]. Dose = 5 mL × 3 kg BW.
- Group 4 (Probiotic Group): Orally administered with Biolact. The probiotic was given twice daily (one sachet/interval, mixed with the milk of their dams) containing 5 × 10^9^ CFU. The administered dose was based on the manufactured product.

### Pharmacokinetic pathways of therapy administration

Each therapy used in the current study followed a distinct pharmacokinetic pathway determined by its route of administration and molecular characteristics. Camel milk and probiotics were administered orally, simply passing to the gastrointestinal tract where they enhance immunity and neutralize pathogens. In our study, the average IgG evel in camel milk was estimated at 3.64 μg/mL, and the IgA level at 2.61 μg/mL. Lactoferrin is another bioactive component present in camel milk, with an estimated concentration of 0.229 ± 0.135 mg/mL [32]. Once milk reaches the lambs’ intestines, enterocytes (absorptive cells) can absorb antibodies, which are then transferred to the bloodstream via lymphatic or portal circulation. Therefore, lambs’ passive immunity will be enhanced against *E. coli* infection. The probiotic group of lambs was fed a diet mainly consisting of non-pathogenic bacterial species, including Lactobacillus spp., *Streptococcus thermophilus*, and *Bifidobacterium* spp. These bacteria tend to colonize the intestine and compete with *E. coli* through adhesion inhibition, enhanced immunity, and increased intestinal acidity. The selected antibiotics and camel-derived plasma were administered parenterally to the respective groups, bypassing the gastrointestinal tract via local injection and then diffusing into the bloodstream. Gentamicin and ciprofloxacin were administered intramuscularly. They can be distributed primarily through the extracellular compartment before entering the systemic circulation. Antibiotics have low affinity for plasma proteins and are freely distributed in the blood due to their high polarity; however, they diffuse passively into the interstitial spaces until they reach satisfactory levels. The bioavailability of gentamicin and ciprofloxacin was estimated at 90%–100% [33, 34]. The mechanism of action of gentamicin and ciprofloxacin aimed to inhibit bacterial DNA replication and transcription. For example, gentamicin can target the 30S subunit of bacterial ribosomes and interfere with messenger RNA (mRNA) translation [35].

**Figure 2:**
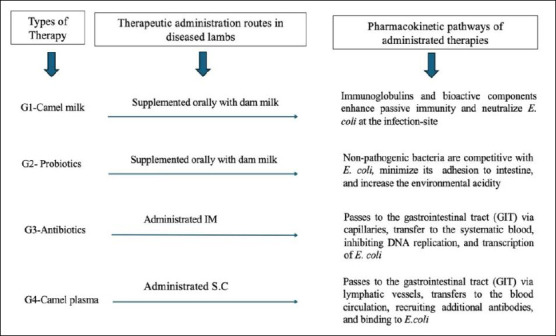
Schematic of the therapies used in neonatal lambs with colibacillosis.

Camel plasma was administered subcutaneously to neonate lambs, and due to its high protein and antibody composition, it is absorbed primarily through lymphatic vessels and to a lesser extent by blood capillaries, after first dispersing within the interstitial fluid. Finally, they are conveyed through the bloodstream to high-perfusion tissues (liver and lungs) and mucosa-associated lymphoid tissue (digestive and respiratory tracts), after which they target foreign pathogens. Camel plasma is rich in immunoglobulins, with an average IgG concentration estimated in this study at 3.55 μg/mL and IgA at 4.24 μg/mL. Figure 2 briefly outlines the framework for the four treatment types and their pharmacodynamic effects on *E. coli* infection.

### Measurement of hematobiochemical parameters

The samples were sent to the laboratory for full blood analysis, including red blood cell counts, WBC, platelet counts, hemoglobin, and hematocrit. The Veterinary Hematology Auto-Analyzer (Exigo H400, Boule Medical AB, Stockholm, Sweden) was used to quantify the results of the samples. This medical device is calibrated and certified annually by Boule Medical AB. A comprehensive metabolic panel was measured using the Veterinary Chemistry Analyzer (Mindray, Shenzhen, China) and included total protein, albumin, globulin, total bilirubin, blood urea nitrogen, creatinine, glucose, total cholesterol, Aspartate Aminotransferase, and Alanine Aminotransferase. The biochemistry device also undergoes yearly calibration, which is certified annually by Mindray Healthcare Company.

### Molecular detection of samples

Bacterial colonies were individually cultured in nutrient broth and incubated at 37°C for 24 h. Then, 2 mL of each culture was centrifuged at 7,826 × *g* for 2 min to pellet the cells. Genomic DNA was extracted from the pellets using the Wizard Genomic DNA Purification Kit (Promega, Madison, WI, USA) according to the manufacturer’s instructions. The concentration of the extracted DNA was measured in ng/μL and its purity was assessed using a NanoDrop spectrophotometer by determining the A260/A280 absorbance ratio, with samples showing ratios of approximately 1.8–2.0 considered suitable for PCR analysis. The DNA samples were stored at −20°C until further use and diluted to a working concentration of approximately 50 ng/μL before amplification. PCR was performed to amplify a specific region of the *uidA* gene for the detection of *E. coli* using a previously reported primer [36]: forward primer (5′-TGGTAATTACCGACGAAAACGGC-3′) and reverse primer (5′-ACGCG TG GTTACAGTCTTGCG-3′). Each PCR reaction was performed in a total volume of 25 μL containing 12.5 μL of 2× GoTaq Hot Start Green Master Mix (Promega, Madison, WI, USA), 1.0 μL each of forward and reverse primers (10 μM), 2.0 μL of template DNA, and 8.5 μL of nuclease-free water. Amplification was conducted in a SimpliAmp Thermal Cycler with an initial denaturation at 95°C for 5 min, followed by 35 cycles of denaturation at 95°C for 30 s, annealing at 54.5°C for 30 s, and extension at 72°C for 45 s, with a final extension at 72°C for 7 min. The PCR products were separated on a 1.5% agarose gel prepared in 1× TAE buffer, stained with ethidium bromide, and visualized under ultraviolet light using a GeneRuler DNA ladder as a molecular size marker.

### ELISA for IgA and IgG testing

**Figure 3:**
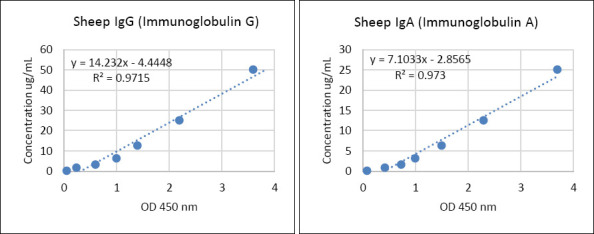
Standard curves of immunoglobulin IgG and IgA concentrations.

Serum IgG and IgA concentrations in pre- and post-treatment lambs were assessed using a sandwich ELISA kit (ELK Biotechnology, Wuhan, China) according to the manufacturer’s instructions. All ELISA measurements were performed using technical duplicates for each biological sample (individual lamb serum). The standard and control samples were run in duplicate on each plate to ensure assay accuracy and reproducibility. Undiluted serum samples (100 μL) were initially assayed. Samples with optical density (OD) values exceeding the standard curve range were diluted 1:5, reassayed using freshly prepared standard curves, and corrected using the appropriate dilution factor. Standard curves were generated for each ELISA plate by plotting sheep IgG/IgA concentration against absorbance values. Assay validity was accepted when the coefficient of determination (R²) was ≥ 0.97, indicating a strong linear relationship between absorbance and Ig concentration. Duplicate readings for the standards, controls, and samples were averaged, and the mean zero-standard OD was subtracted prior to analysis. Data analysis and curve fitting were performed using CurveExpert software version 1.40 (https://www.abbkine. com/draw-elisa-standard-curve/) and Microsoft Excel 365 (Microsoft, Washington, USA). Following the completion of the assay, the enzyme–substrate reaction was terminated by the addition of sulfuric acid stop solution, and absorbance was measured at 450 nm (±10 nm) using a microplate reader. The IgG and IgA concentrations were calculated by comparing the sample OD values to the corresponding standard curves. The assay detection range for sheep IgG is 1.57–100 μg/mL, with a sensitivity of 0.55 μg/mL, while the detection range of IgA is 0.79–50 μg/mL and its sensitivity is 0.34 μg/mL. The intra-assay and inter-assay coefficients of variation were <8% and <10%, respectively. Serum samples were processed immediately or stored at −20°C and were not subjected to repeated freeze–thaw cycles prior to analysis. Figure 3 shows the standard curve of IgG and IgA.

### Statistical analysis

Data were entered into an Excel sheet and analyzed using JAMOVI (version 2.3, Sydney, Australia). One-way analysis of variance was conducted, and a post-hoc test (Tukey’s honestly significant difference) was used to determine which specific group means differ significantly from each other or the comparison group (p < 0.05). The mean and standard deviation for each variable is reported in the tables. A normality test (Shapiro–Wilk) was performed to diagnose any violation in the data (a low p-value indicates a violation).

## RESULTS

### Identification of *E. coli* and analysis of milk components

**Figure 4:**
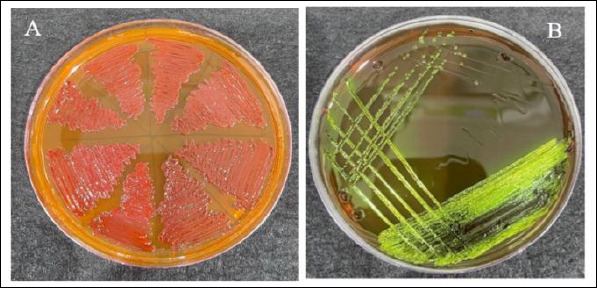
(A) Growth of *Escherichia coli* colonies on MacConkey agar, typically reddish-pink. (B) *E. coli* colonies on Eosin Methylene Blue agar with a metallic green sheen.

**Figure 5:**
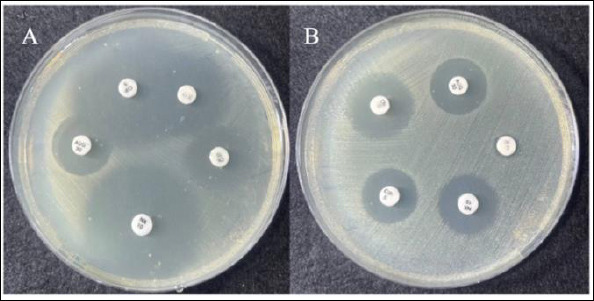
Disk diffusion (Kirby–Bauer) antimicrobial susceptibility testing of *Escherichia coli* showing zones of inhibition for amoxicillin–clavulanate (AUG, 30 μg), ciprofloxacin (CIP, 5 μg), chloramphenicol (C, 30 μg), gentamicin (CN, 10 μg), and norfloxacin (NX, 10 μg). The zone in the disk diffusion test (A) of NX (28–28 mm), AUG (12–12 mm), CIP (26–26 mm), C (15–15 mm), and CN (14–14 mm). The zone diameters in the disk diffusion (B), AUG and NX (10–10 mm), CN (12–12 mm), CIP (9–9 mm).

A typical morphological characteristic of the *E. coli* colonies on MacConkey agar was a distinctive pink color, while growth on eosin methylene blue (EMB) agar was a metallic green sheen (Figure 4). All these results are from lactose fermentation. According to the antibiotic sensitivity results, *E. coli* was sensitive to all tested antibiotics, with the degree values varying (Figure 5). Ciprofloxacin, norfloxacin, and chloramphenicol showed a susceptibility rate of 40%, while gentamicin (60%) and amoxicillin (50%) were classified as intermediate/susceptibility-dose dependent for microbial sensitivity.

**Figure 6:**
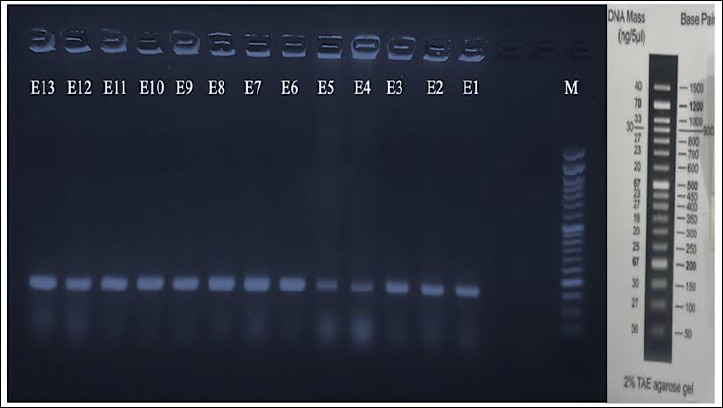
Gel electrophoresis showing the amplicon of the 162 bp (*uidA* gene) of *Escherichia coli* isolates from infected lamb. The DNA marker (M) and the 13 samples are shown as distinct bands. The first 6 bands represent selected positive samples of initial diagnosis from diarrheic lambs, while the remaining 7 bands represent selected positive samples from the treatment groups.

PCR amplification of the target gene (*uidA*) in both groups yielded a distinct amplicon of approximately 162 bp, as visualized by electrophoresis on a 1.5% agarose gel (Figure 6). The band size was determined by comparison with a standard molecular DNA ladder and precisely corresponded to the expected product size for the specific gene region of *E. coli*. This result confirms the successful and specific amplification of the target gene, supporting the molecular identification of *E. coli* from diarrheic lambs.

**Figure 7:**
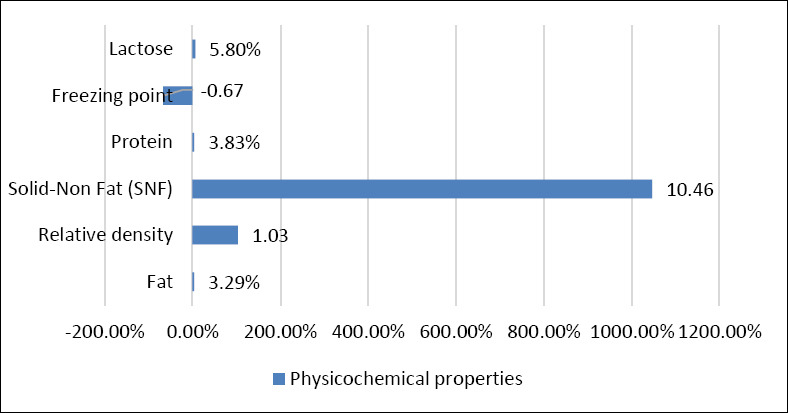
Shows average (μ) of the physicochemical properties of camel milk (lactose, freezing point, protein, Solid-not fat, relative density, and fat).

The physicochemical analysis of camel milk varied among the parameters tested (Figure 7). The fat content was estimated to be 3.29%, the mean relative density was 1.03, the mean SNF content was 10.46%, the mean protein content was 3.83%, the mean freezing point was -0.67°C, and the mean lactose content was 5.80%.

### Treatment and safety monitoring groups

All animals were monitored intensively over a 5-day period, including assessment of rectal temperature, hydration status, fecal consistency, appetite, and health activity. Lambs showed varying degrees of improvement throughout the treatment period, with no evidence of treatment-related complications. All animal groups exhibited normal temperature during treatments (38°C–39°C). Lambs treated with camel milk and probiotic showed a moderate improvement in appetite behavior and low hydration (Table 1). Animals treated with antibiotics showed little improvement in all clinical parameters. Fecal consistency changed to semi-solid in camel milk- and antibiotic-treated animals. A significant improvement was noticed in the group treated with camel plasma after 2 days of injection. Their hydration and appetite status were remarkably improved, and their fecal consistency returned to normal (soft to pasty). However, no signs of local swelling, allergic responses, anaphylaxis, or delayed hypersensitivity were observed in the plasma-treated group.

**Table 1:** Clinical observations of lambs after treatment in each allocated group.

Treatment	Temperature	Appetite	Hydration status	Fecal consistency	Activity
Camel milk	Normal	Middle-improved	Low-improved	Semi-solid	Good
Probiotics	Normal	Middle-improved	Low-improved	Soft to pasty	Good
Antibiotics	Normal	Low-improved	Low-improved	Semi-solid	Weak
Camel plasma	Normal	Highly improved	Highly improved	Soft to pasty	Excellence

### Hematobiochemical analyses

Several hematological and biochemical parameters were reported to be compared between the treatment and infected groups of lambs in this study (Table 2). We found that the WBC level was significantly reduced by all treatment groups, with a p-value estimated to be < 0.05. The lymphocyte and monocyte levels did not change significantly across all treatment groups, whereas granulocyte levels decreased significantly in the treatment groups (p-value < 0.05) compared with the infected group. Red blood cells (RBC), hematocrit, platelets, and hemoglobin (HB) remained stable in all groups. In addition, the levels of mean corpuscular volume (MCV) and mean corpuscular hemoglobin (MCH) did not change significantly compared with the infected animals. MCH concentration (MCHC) were relatively increased in lambs treated with camel milk, camel plasma, and probiotics.

**Table 2:** Comparison of the hematological parameters in lambs treated with the four types of treatment. Each group included 5 neonate lambs treated for 5 days.

Parameters	Infected	Antibiotic	Camel milk	Camel plasma	Probiotics	Reference range
WBC (10^9^/L)	14.78 ± 3.60	7.40 ± 1.05*	8.96 ± 2.03*	7.52 ± 0.95*	6.70 ± 0.98*	4.0–12.0
Lymphocyte (10^9^/L)	3.04 ± 0.36	2.94 ± 0.41	2.46 ± 0.27	2.92 ± 0.57	2.64 ± 0.26	2.0–9.0
Monocytes (10^9^/L)	0.68 ± 0.13	0.62 ± 0.16	0.56 ± 0.23	0.52 ± 0.13	0.36 ± 0.20	0.0–0.8
Granulocyte (10^9^/L)	10.98 ± 3.26	3.70 ± 0.97*	5.92 ± 1.76*	4.98 ± 0.82*	2.70 ± 1.19*	0.7–6.0
RBC (10¹²/L)	10.35 ± 0.60	9.28 ± 1.32	9.60 ± 0.72	10.49 ± 1.33	10.80 ± 1.28	9.0–15.0
HCT (%)	31.28 ± 1.24	31.38 ± 5.97	28.80 ± 1.84	37.76 ± 9.50	35.42 ± 5.73	27.0–45.0
Platelet (10^9^/L)	621 ± 126	749 ± 146	822 ± 132	640 ± 168	793 ± 122	200–800
HGB (g/dL)	11.34 ± 0.71	11.70 ± 0.90	11.30 ± 0.74	11.26 ± 0.65	11.26 ± 0.81	9.0–15.0
MCH (pg)	11.70 ± 0.15	12.74 ± 0.89	11.94 ± 0.60	12.68 ± 0.90	11.04 ± 1.00	8.0–12.0
MCV (fL)	30.32 ± 0.38	34.68 ± 2.42	30.54 ± 2.82	31.92 ± 2.28	27.20 ± 2.70	25.0–40.0
MCHC (g/dL)	38.60 ± 0.93	37.64 ± 0.64	39.06 ± 1.73	39.14 ± 0.74	40.40 ± 1.34	31.0–34.0

WBC = White blood cell, RBC = Red blood cell, HCT = Hematocrit, HGB = Hemoglobin, MCH = Mean corpuscular hemoglobin, MCV = Mean corpuscular volume, MCHC = Mean corpuscular hemoglobin concentration.

* Considered significant if p ≤ 0.05.

The biochemical findings revealed only slight increases in the concentrations of some proteins and liver enzymes across the treatment groups (Table 3). Albumin, total protein, globulin, and creatinine were moderately increased in camel plasma-treated animals. While these levels did not change in the animals fed antibiotic and camel milk, unlike the group fed with probiotic, the total protein (34.48 ± 14.35 g/L) and globulin (12.98 ± 8.24 g/L) were decreased compared with the infected or control group. There was an increase in the level of aspartate aminotransferase (AST) in the replicates that received camel milk, plasma, and probiotic, with levels estimated at 50.80 ± 24.33 U/L, 80.20 ± 54.15 U/L, and 64.80 ± 13.59 U/L, respectively. The alanine aminotransferase (ALT) enzyme remained unchanged in all groups. Creatinine levels were reduced in animals treated with antibiotics, but were insignificantly elevated in the other treatment groups compared with the infected group.

**Table 3:** Biochemical parameters (blood protein, total cholesterol, and glucose) and liver enzymes in four groups of the diarrheic lambs. Samples were obtained from infected (n = 5) and post-treatment (n = 20) animals.

Parameters	Infected	Antibiotic	Camel milk	Camel plasma	Probiotic	Reference range
Albumin (g/L)	27.98 ± 1.66	25.90 ± 1.75	27.52 ± 4.94	29.10 ± 4.08	21.50 ± 7.28	20–49
Total protein (g/L)	48.12 ± 5.39	45.24 ± 6.87	47.46 ± 6.82	52.72 ± 11.97	34.48 ± 14.35	39–99
Globulin (g/L)	20.14 ± 5.48	19.34 ± 5.82	19.94 ± 5.51	23.62 ± 8.24	12.98 ± 8.24	13–74
Glucose (mmol/L)	5.38 ± 0.59	5.47 ± 0.42	5.87 ± 1.70	4.43 ± 1.27	4.76 ± 2.04	2.6–17.5
BUN (mmol/L)	4.38 ± 0.73	4.45 ± 0.55	3.82 ± 0.98	4.58 ± 1.24	4.22 ± 1.27	3.9–17.5
T. Cholesterol (mmol/L)	1.92 ± 0.44	2.48 ± 0.42	2.32 ± 0.44	1.86 ± 0.30	2.07 ± 0.47	0.2–6
ALT (U/L)	11.16 ± 1.11	12.00 ± 0.00	11.40 ± 0.89	12.00 ± 0.70	13.00 ± 1.58	0–70
AST (U/L)	44.60 ± 5.32	41.80 ± 7.12	50.80 ± 24.33	80.20 ± 54.15	64.80 ± 13.59	0–530
T. Bil (μmol/L)	5.36 ± 0.76	4.82 ± 0.17	5.01 ± 0.45	4.98 ± 0.69	4.50 ± 1.55	0–36
Creatinine (μmol/L)	43.20 ± 9.88	23.80 ± 1.92	60.40 ± 37.70	54.20 ± 80.46	55.60 ± 15.32	45–290

BUN = Blood urea nitrogen, T. Cholesterol = Total cholesterol, ALT = Alanine aminotransferase, AST = Aspartate aminotransferase, T. Bilirubin = Total Bilirubin.

### ELISA results for immunoglobulin (IgA and IgG) testing

The concentrations of immunoglobulin IgA and IgG were measured among the four treatment groups. The average IgG level in the infected lambs was calculated as 5.14 μg/mL (Table 4). The IgG levels were significantly stabilized (2 μg/mL) in the animals that received camel milk, camel plasma, and probiotics, with a p-value of 0.001. The IgA concentration was significantly normalized in lambs treated with camel plasma and probiotics (p-values <0.001 and 0.0018, respectively). A significant normalization of the IgA level (declined from 2.03 ± 0.43 to 0.42 ± 0.15 μg/mL) was observed in lambs treated with camel plasma (p-value < 0.05) compared with other treatment groups.

**Table 4:** ELISA assessment of IgG and IgA levels from serums of pre- and post-treatment lambs.

Treatment group	IgG level (μg/mL)		IgA level (μg/mL)	

	Pre-treatment	Post-treatment	Pre-treatment	Post-treatment
Antibiotics	5.15 ± 0.13	3.26 ± 0.36*	2.10 ± 0.21	2.12 ± 0.43
Camel milk	5.54 ± 0.55	1.93 ± 0.19*	2.41 ± 0.15	2.43 ± 0.14
Camel plasma	5.26 ± 0.49	2.15 ± 0.40*	2.03 ± 0.43	0.42 ± 0.15*
Probiotics	5.72 ± 0.51	2.18 ± 0.30*	2.20 ± 0.11	1.42 ± 0.16*

* Considered significant if p ≤ 0.05.

## DISCUSSION

### Novelty and contributions of the study

The novelty of this research may help mitigate livestock losses from bacterial infections, provide alternative antibiotic sources, and enhance FPT in neonates. Our study introduces a new approach to treatment using camel plasma and camel milk, and evaluates the efficacy of selective antibiotics and probiotics in neonatal lambs infected with *E. coli*. The utilization of camel plasma as a therapeutic approach for *E. coli* infection in ruminants has never been tested systematically before. However, the high mortality rate in neonate lambs affected by gastrointestinal infection is largely associated with the FPI transfer. Other risk factors, including a malnutrition state of ewes, unvaccinated pregnant ewes, and toxigenic virulence of the pathogen, may also be attributed to lamb death or severe morbidity [37].

### Post-treatment assessments and methodological considerations

The findings of this study indicated that all treated groups showed varying degrees of changes in hematobiochemical indices. The post-treatment assessments were intentionally conducted over a short period to capture the study subjects’ acute physiological responses. This methodological approach is consistent with recent experimental studies that have evaluated rapid hematological and biochemical changes following pharmaceutical or nutritional interventions [38–40]. According to literature reviews, measuring blood parameters is a crucial indicator for disease infection response in immunocompromised animals [41, 42]. However, camel products (milk and plasma), particularly plasma, exhibit excellent therapeutic efficacy by constraining the inflammatory response and modulating serum protein profiles.

### Administration route and safety of camel plasma

For the first time, camel plasma was administered subcutaneously to diarrheic lambs, which is assumed to be a safer route and can be facilitated by physiological processes that ultimately target the infection at the mucosal-associated site. Administration of camel plasma via intravenous injection may be risky and may lead to blood clot formation due to the presence of clotting factor VIII in camel plasma [43]. The study also highlights that the immunoglobulins of lambs (IgA and IgG) were stabilized or normalized by all treatment types, and more significantly in replicates treated with camel plasma. The study found that camel plasma is an effective therapy for colibacillosis and a potential immunological enhancer for individuals with depleted passive immunity.

### Prevalence and transmission of *E. coli* infection

In the present study, *E. coli* was identified in all examined animals, indicating that this pathogen plays a major role in neonate lamb infection in the studied populations. Infection transmission in lamb populations occurs due to environmental contamination, poor hygiene, and ecological factors [44, 45]. In addition, high susceptibility to *E. coli* infection in lamb is due to immature immunity and insufficient colostrum intake [46]. *E. coli* infection rates vary across regions in Iraq. In Nineveh, a province located in the north of Iraq, an epidemiological study found that the prevalence of *E. coli* was estimated to be 60%, and two toxins related to Shiga were identified in 91 samples isolated from suckling lambs, including stx1 and stx2 [47]. Other surveyed studies from the Duhok and Baghdad provinces indicated that the prevalence of *E. coli* in sheep was 3% and 78%, respectively [48, 49]. The mortality rate in neonate lambs may be increased by *E. coli* infection due to toxins, severe dehydration, and antibiotic resistance. Protecting healthier lambs from *E. coli* infection can be achieved through several control measures, including: vaccinating a pregnant ewe twice during the gestational period; receiving newborn lamb adequate amount of colostrum during 28–48 h of life; reducing environmental contamination by isolating diseased animals, and supplying probiotics to young sheep to enhance their passive immunity [6, 50].

### Antimicrobial susceptibility patterns

The antimicrobial susceptibility test revealed that *E. coli* isolates showed 40% sensitivity to ciprofloxacin, norfloxacin, and chloramphenicol, whereas gentamicin showed 60% sensitivity, evaluated as intermediate sensitivity/susceptibility-dose dependent. Comparable results were also reported in previous research studies regarding variation in antibiotic resistance. El-Nady *et al*. [51] reported that 62 of 95 diarrheic lambs in Egypt were positive for *E. coli* and were completely resistant to macrolides and tetracyclines due to the presence of beta-lactam resistance genes. In the Northwest of China, an investigation of 500 *E. coli* isolates found that approximately 11 antimicrobial drugs, including sulfisoxazole, florfenicol, and tetracyclines, were resistant, with 80% of the isolates encoding the *etrA* resistance gene [52]. Biofilm formation, genetic variation, and environmental factors may significantly influence the antibiotic resistance of *E. coli*. Variation and degree of antibiotic sensitivity in *E. coli* are associated with diverse genes located on chromosomes or plasmids [53]. Overcoming these challenges and promoting animal welfare can be achieved through the use of non-antibiotic alternatives.

### Comparative treatment effects on clinical and hematological parameters

The competitive treatments approach was adopted, using four treatment types for symptomatic diarrheic lambs diagnosed with *E. coli*. Following 5 consecutive days of treatment, the diseased lambs received the selective antibiotic, camel milk, camel plasma, and probiotic supplement. Then, hematobiochemical parameters were analyzed to explore the effects on each comparison group. All treatment groups showed a reduction in leucocyte levels. The total WBC and granulocyte counts were significantly decreased in all treated animals compared with infected animals. These findings are consistent with those of a clinical study by Hassan *et al*. [54], who recommended ciprofloxacin and gentamicin for treatment of *E. coli* infections in young lambs, with improvements in WBC and RBC levels observed after 6 days of treatment. Our study also aligns with a study by Anyika *et al*. [55], who fed neonate lambs probiotic starters (*Lactobacillus acidophilus*, *B. pumilus*, *B. subtilis*, and *B. licheniformis*), resulting in *E. coli* counts of 2 × 10³ CFU/g after 6 weeks of regular treatments. A study from Germany indicated that the administration of the non-pathogenic bacteria (*E. coli* strain Nissle 1917) for calves for the first 2 weeks of life reduced the incidence of diarrhea to 26.5% [56]. This explains how probiotics and specific antibiotics may positively influence the gut microbiota and enhance the immunity of newborn lambs [57, 58].

### Immunomodulatory effects of camel milk and plasma

In addition, the leukocyte counts improved following treatment with camel milk and plasma. This finding is consistent with a study by Hassaneen *et al*. [59], which indicated the beneficial effects of camel milk in mitigating aflatoxin B1 (AFB1) toxicity in laboratory rats. That study reported significant improvements in liver function parameters, oxidative stress, and counts increasing from 8.08 × 10³ μ/L to 10.10 × 10³ μ/L. These results are attributed to the immunomodulatory properties of camel milk, which contains abundant exosomes and lactoferrin mRNA and significantly downregulates interferon-gamma (IFN-γ) expression and reduces T helper cell activity [19, 60]. On the other hand, the erythrocyte indices (RBC, HB, MCH, and MCV) did not change markedly in any of the treated groups, which is consistent with the findings of Ayala-Monter *et al*. [61]. The MCHC levels showed an insignificant increase in lambs treated with probiotics, suggesting enhanced gut absorption and reduced oxidative stress. Moreover, feeding probiotics to animals promotes the intake of micronutrients such as vitamin B12, calcium, folate, iron, and zinc, which are necessary for erythropoiesis [62]. An experimental study by Asadi *et al*. [63] also reported a marked improvement in MCHC (from 18.58 in the control group to 23.12 mmol/L) and other hematological parameters in suckling lambs supplemented with organic iron (25 mg/d). This highlights the importance of adequate micronutrients in dam’s milk to avoid anemia in neonate lambs. Animal age, farmer management, and environmental variables are attributable factors linked to the health and welfare of domestic animals [64, 65].

### Biochemical responses and liver enzyme changes

Based on the biochemical results, serum protein parameters, including albumin, total protein, globulin, and creatinine, were moderately elevated in camel plasma, although these changes remained within the normal range (Table 3). This effect may be attributed to the enrichment of camel plasma with several micronutrients and bioactive compounds, including calcium (Ca), phosphorus (P), magnesium (Mg), and copper (Cu), which play vital roles in maintaining homeostasis and overall health [66, 67]. These elements were estimated to be much higher than those in bovine or sheep plasma [68]. Another finding from the present study is that AST was fairly elevated in animals treated with camel milk (50.80 ± 24.33 U/L), camel plasma (80.20 ± 54.15 U/L), and probiotics (64.80 ± 13.59 U/L). A plausible explanation for this result is that cytokine production may be stimulated and gluconeogenesis enhanced, which in turn activates the cytoplasmic form of AST in muscle and heart tissues rather than indicating hepatocellular damage. A similar outcome was reported by Helal *et al*. [69], who observed a significant increase in AST levels in diabetic model rats supplemented with camel milk for 30 days. Therefore, additional research is needed to better understand the effects of camel products, particularly camel plasma, through longitudinal monitoring of liver enzymes and serum protein parameters in animals over several days following treatment.

### Immunoglobulin stabilization and passive immunity enhancement

Camel plasma improved clinical symptoms and immunological parameters, confirming the ability of plasma antibodies to neutralize the pathogen in lambs. Serum IgG concentrations typically become higher during the first day after birth because of colostrum intake, and their level gradually declines between 14 and 60 days of age [46]. In addition, there is a strong correlation between γ-glutamyltransferase (GGT) and IgG levels in lambs; when a GGT cut-off below 500 IU/L is used to determine failure of passive immunity, this increases susceptibility to illness or death [70]. Our study demonstrated that IgG levels were rapidly alleviated or stabilized from 5 μg/mL to around 2 μg/mL in animals following treatment with camel milk, camel plasma, and probiotics. Camel milk contains natural bioactive compounds, whereas probiotics comprise beneficial microbes. However, each treatment independently enhances passive immunity and modulates reduced immune responses [71, 72]. Immunoglobulin levels, particularly IgA (0.42 ± 0.15 μg/mL), were normalized following camel plasma administration, indicating the high efficacy of camel plasma as a non-antibiotic therapeutic approach and its ability to robustly transfer passive immunity (FPT). Plasma transfusion is a common practice in veterinary medicine, and both donors and recipients should be cross-matched for blood type. Other studies have demonstrated that although plasma-derived IgG administration in neonatal calves results in a significant increase in serum IgG concentrations within the first 24 h, this intervention does not reduce morbidity or mortality rates among newborn calves [73–75]. However, camel serum and plasma contain heavy-chain antibodies (HCAbs) composed of single-domain variable regions known as nanobodies or VHH (Variable domain of heavy-chain), which are characterized by their small size (15 kDa), high stability, and strong affinity [76]. They differ in size and structure from conventional IgG, which have medical benefits for the treatment of viral diseases and cancer [77]. Although HCAbs were not assessed practically in this experimental study, it is theoretically assumed to contribute to treatment effects by plasma. The bioavailability of immunoglobulins and peptides in camel plasma is lower than that of antibiotics; however, their mode of action is more efficient, as they actively strengthen passive immunity and bind to *E. coli* with high affinity, resulting in rapid neutralization and infection control.

## CONCLUSION

This pilot study demonstrated that camel plasma serves as a highly effective non-antibiotic therapeutic option for *E. coli*-induced colibacillosis in neonatal lambs. Compared with antibiotics, camel milk, and probiotics, camel plasma treatment resulted in the most rapid and pronounced clinical improvements, including markedly enhanced appetite, activity, hydration status, and fecal consistency (returning to soft/pasty within 2 days), with no adverse reactions such as local swelling, anaphylaxis, or hypersensitivity. Hematologically, all treatments significantly reduced WBC counts (from 14.78 ± 3.60 to approximately 7 × 10^9^/L) and granulocyte counts (p < 0.05), while biochemical parameters (albumin, total protein, globulin, creatinine) showed moderate increases in the camel plasma group. Immunologically, camel plasma achieved the most significant normalization of serum IgA levels (declined from 2.03 ± 0.43 to 0.42 ± 0.15 μg/mL; p < 0.05) and stabilization of IgG concentrations (approximately 2 μg/mL; p = 0.001), outperforming other interventions in supporting passive immunity.

These findings carry important practical implications for small ruminant farming in resource-limited and arid regions. Camel plasma offers a locally accessible, sustainable alternative to conventional antibiotics, with the potential to reduce antimicrobial use, mitigate the spread of AMR, enhance farm biosecurity, and lower the risk of zoonotic transmission of *E. coli* to humans. By leveraging camel-derived products already available in many pastoral communities, this approach aligns with One Health principles, promoting animal welfare, economic sustainability, and public health safety without reliance on imported pharmaceuticals or complex infrastructure.

The major strengths of the study include its field-based design under real farm conditions, a direct head-to-head comparison of four therapeutic modalities in naturally infected lambs, a comprehensive evaluation of clinical, hematobiochemical, and immunological endpoints, and the innovative use of subcutaneous camel plasma administration as a novel, safer route compared with intravenous administration. The study also benefited from rigorous ethical oversight, strict animal welfare protocols, and molecular confirmation of *E. coli* via amplification of the *uidA* gene.

Limitations include the small sample size per group (n = 5), the short 5-day treatment and observation period (which captured acute responses but not long-term outcomes), the pilot nature of the trial, and the lack of assessment of plasma biosafety, colostrum quality, maternal antibody status, and heavy-chain antibody (HCAbs/nanobodies) contributions in camel plasma. Convenient sampling and regional specificity (Safwan subdistrict, Basrah) may limit generalizability.

Future research should prioritize larger-scale, multicenter randomized controlled trials with extended follow-up (3–6 months) to evaluate long-term survival, growth performance, recurrence rates, and cost-effectiveness. Longitudinal monitoring of liver enzymes, serum protein profiles, and AMR patterns, together with detailed characterization of camel plasma immunoglobulins (including HCAbs/VHH domains), will be essential. Investigating optimal dosing regimens, storage stability, and standardized production protocols for camel plasma will support its practical adoption as a scalable, non-antibiotic intervention in veterinary medicine.

In conclusion, camel plasma emerges as a promising, immunity-enhancing, non-antibiotic therapy capable of effectively managing neonatal colibacillosis while addressing the pressing global challenge of AMR. By harnessing the unique immunological properties of camel plasma, this approach not only improves lamb health and survival but also contributes to sustainable livestock production and One Health goals in challenging environments. Further validation through larger studies is warranted to translate these encouraging pilot findings into widespread clinical and field application.

## DATA AVAILABILITY

The data generated from this study are available based on a reasonable request.

## AUTHORS’ CONTRIBUTIONS

HRT, NAK, and MFA: conceived the idea of the study. HRT: carried out the research fieldwork. NAK and MFA: Supervision, data analyses, and writing, review, and editing. All authors have reviewed and approved the final version of the manuscript.
